# Alteration of the DNA Methylation Signature of Renal Erythropoietin-Producing Cells Governs the Sensitivity to Drugs Targeting the Hypoxia-Response Pathway in Kidney Disease Progression

**DOI:** 10.3389/fgene.2019.01134

**Published:** 2019-11-13

**Authors:** Koji Sato, Naonori Kumagai, Norio Suzuki

**Affiliations:** ^1^Division of Oxygen Biology, United Centers for Advanced Research and Translational Medicine, Tohoku University Graduate School of Medicine, Sendai, Japan; ^2^Division of Nephrology, Endocrinology, and Vascular Medicine, Tohoku University Graduate School of Medicine, Sendai, Japan; ^3^Department of Pediatrics, School of Medicine, Fujita Health University, Toyoake, Japan

**Keywords:** chronic kidney disease, DNA methylation, fibrosis, hypoxia, renal anemia, urine exfoliated cells

## Abstract

Chronic kidney disease (CKD) affects more than 10% of the population worldwide and burdens citizens with heavy medical expenses in many countries. Because a vital erythroid growth factor, erythropoietin (EPO), is secreted from renal interstitial fibroblasts [renal EPO-producing (REP) cells], anemia arises as a major complication of CKD. We determined that hypoxia-inducible factor 2α (HIF2α), which is inactivated by HIF-prolyl hydroxylase domain-containing proteins (PHDs) in an oxygen-dependent manner, tightly regulates EPO production in REP cells at the gene transcription level to maintain oxygen homeostasis. HIF2α-mediated disassembly of the nucleosome in the *EPO* gene is also involved in hypoxia-inducible EPO production. In renal anemia patients, anemic and pathological hypoxia is ineffective toward EPO induction due to the inappropriate over-activation of PHDs in REP cells transformed into myofibroblasts (MF-REP cells) due to kidney damage. Accordingly, PHD inhibitory compounds are being developed for the treatment of renal anemia. However, our studies have demonstrated that the promoter regions of the genes encoding EPO and HIF2α are highly methylated in MF-REP cells, and the expression of these genes is epigenetically silenced with CKD progression. This finding notably indicates that the efficacy of PHD inhibitors depends on the CKD stage of each patient. In addition, a strategy for harvesting renal cells, including REP cells from the urine of patients, is proposed to identify plausible biomarkers for CKD and to develop personalized precision medicine against CKD by a non-invasive strategy.

## Renal Anemia

Currently, over 10% of the population worldwide suffers from chronic kidney disease (CKD), which is characterized by kidney dysfunction and/or proteinuria that persists for more than 3 months (Levery et al., 2005). A gradual decline in kidney function results in sclerotic lesions, cardiovascular disease, and mortality (Imai et al., 2009; Hill et al., 2016). While the etiologies of CKD are diverse, ranging from lifestyle-related diseases to autoimmune disorders, CKD progression is commonly accompanied by kidney fibrosis, in which myofibroblasts emerge and proliferate in the renal tubular interstitium (Quaggin and Kapus, 2011). Because kidneys are the major organs producing erythroid growth factor erythropoietin (EPO) in adult mammals (Suzuki, 2015; Hirano and Suzuki, 2019), erythropoiesis is often impaired in CKD patients (Nangaku and Eckardt, 2006). The liver supportively produces EPO under anemic conditions, but hepatic EPO production cannot adequately compensate for renal EPO production in renal anemia patients. In fact, mice lacking renal *EPO* gene expression exhibit severe anemia, although *EPO*-gene expression is induced in their hepatocytes (Yamazaki et al., 2013; Hirano et al., 2017).

Because EPO is required for erythropoiesis, gene-modified mouse lines lacking EPO production exhibit embryonic lethality due to severe anemia (Wu et al., 1995; Yamazaki et al., 2013). Since red blood cells are essential for oxygen delivery to every organ, renal anemia severely decreases the quality of life (QOL) of CKD patients. To maintain oxygen homeostasis, EPO production in the kidney is dramatically enhanced under hypoxic/anemic conditions (Suzuki and Yamamoto, 2016). As CKD progresses, renal EPO production becomes impaired, and renal anemia then develops (Nangaku and Eckardt, 2006; Souma et al., 2015). Intriguingly, recent studies have shown that proper treatment of renal anemia is associated with the prognosis of CKD patients and that the plasma EPO concentration tightly correlates with kidney function and fibrosis (Inomata et al., 1997; Singh et al., 2006; Pfeffer et al., 2009). Thus, plasma EPO is expected to be a plausible biomarker to estimate the CKD grade (Tsubakihara et al., 2015).

For treatment of renal anemia, recombinant human EPO reagents have been used as erythropoiesis-stimulating agents (ESAs) for more than 30 years, and these reagents have dramatically improved the QOL of CKD patients (Jones et al., 2004). However, the invasiveness of subcutaneous ESA injections and the formulation costs of ESAs are problems that need to be solved (Schiller et al., 2008). Additionally, ESAs are frequently ineffective for patients suffering from chronic inflammation because EPO-dependent erythropoiesis is strongly suppressed by high serum concentrations of inflammatory cytokines and hepcidin, which negatively regulates iron usage for hemoglobin (Ganz, 2003; Smrzova et al., 2005; Suzuki et al., 2016; Petrulienė et al., 2017).

## Renal Erythropoietin-Producing Cells

**Figure 1:**
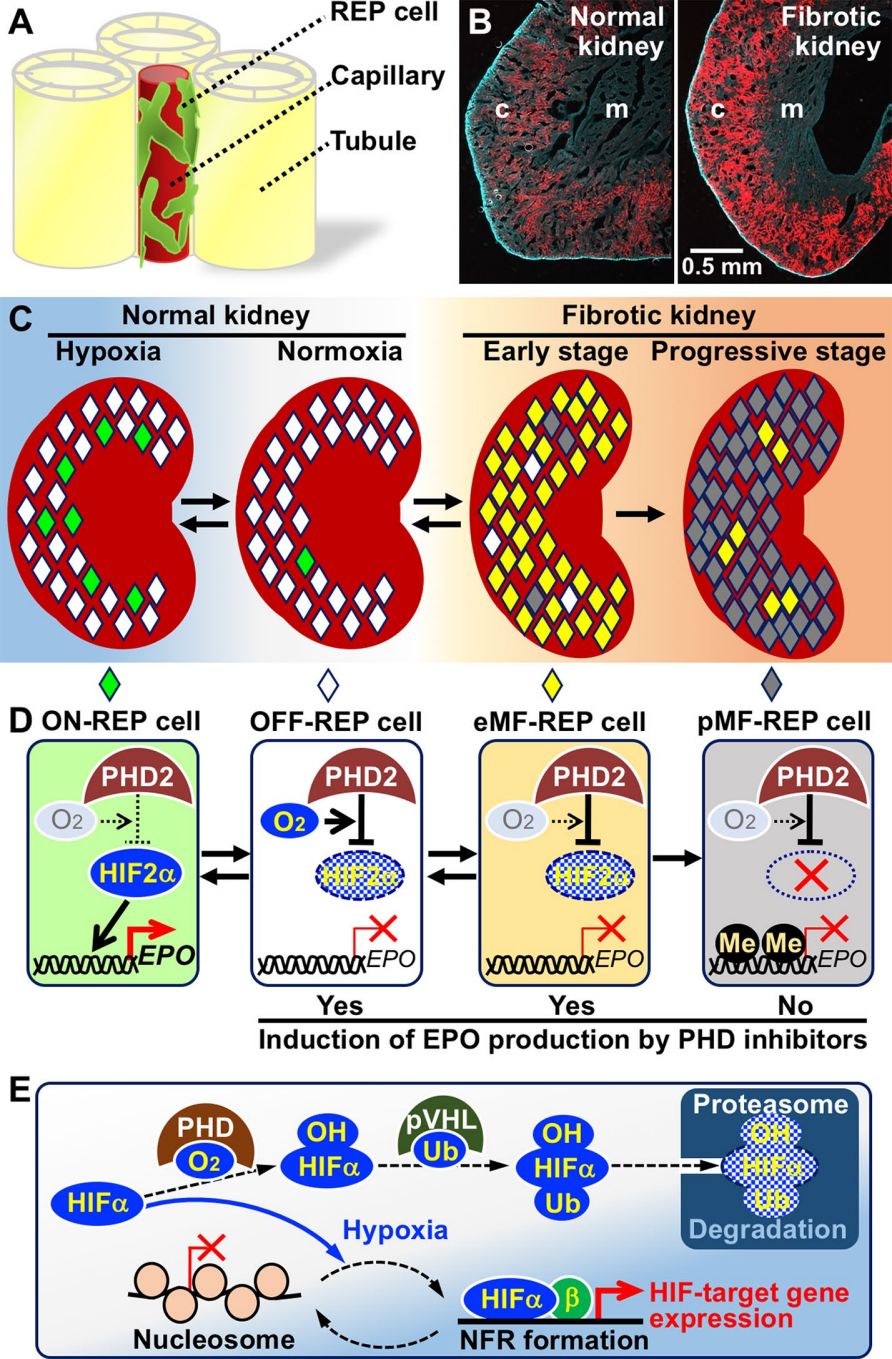
Mechanisms of hypoxia-inducible erythropoietin (EPO) production in renal EPO-producing (REP) cells and failure of EPO production in fibrotic kidney. **(A)** A schema of REP cell localization in the interstitia between renal tubules. REP cells directly associate with capillaries (Souma et al., 2016). **(B)** REP cells (red) distributed to the outer medulla (m) and cortex (c) of a normal healthy kidney (left) are expanded in a fibrotic kidney (right) of a genetically modified mouse line specifically expressing tdTomato fluorescence in REP cells (Yamazaki et al., 2013). **(C)** Distributions of ON-REP (green), OFF-REP (white), early myofibroblast (eMF)-REP (yellow), and progressive MF (pMF)-REP (gray) in normal kidneys and fibrotic kidneys. Note that a small fraction of REP cells produce EPO even under hypoxic conditions (left). **(D)** *EPO*-gene regulation by the PHD2-HIF2α pathway in REP cells and MF-REP cells. In eMF-REP cells (reversibly transformed REP cells), PHD2 over-activation results in inactivation of *EPO*-gene transcription. Therefore, PHD inhibitors may induce EPO production. Because the genes for EPO and HIF2α are epigenetically inactivated due to DNA methylation (Me) in pMF-REP cells (irreversibly transformed REP cells), PHD inhibitors are ineffective. **(E)** Molecular mechanism of hypoxia-inducible transcriptional regulation. HIFα proteins are always synthesized and degraded by the ubiquitin (Ub)-proteasome pathway *via* PHD-mediated hydroxylation (OH) in oxygen-replete cells. In hypoxic cells, PHD is inactivated, and HIFα proteins are stabilized. In some HIF-target gene promoters, HIFα/β complexes mediate the disassembly of nucleosome structures to form nucleosome-free regions under hypoxic conditions.

Using genetically modified mouse lines, we and others demonstrated that the ability to produce EPO is present in most fibroblasts that are positive for CD73 and platelet-derived growth factor receptor β (PDGFRβ) in the interstitium spreading from the cortico-medullary boundary to the renal cortex (**Figures 1A, B**; Maxwell et al., 1993; Pan et al., 2011; Yamazaki et al., 2013). The cells that produce EPO in response to a hypoxic microenvironment are known as REP (renal EPO-producing) cells (Suzuki et al., 2007; Obara et al., 2008). REP cells are fundamentally quiescent in terms of the cell cycle, and EPO production in the majority of REP cells is absent in healthy mice (Souma et al., 2013; Yamazaki et al., 2013). Under hypoxic/anemic conditions, the percentage of “ON-REP cells,” in which EPO production is ongoing, in the total REP cell population is increased. However, only up to 10% of REP cells are ON-REP cells, even under very severe chronic anemia conditions, suggesting that most REP cells are reservoirs (referred to as OFF-REP cells) in preparation for much more severe conditions that require high amounts of EPO (**Figures 1C, D**; Yamazaki et al., 2013; Souma et al., 2015). Thus, the total amount of EPO secretion from a kidney is correlated with the ratio of ON-REP cells to total REP cells, rather than the extent of EPO-production levels in each cell (Eckardt et al., 1993; Obara et al., 2008; Suzuki, 2015). Additionally, these data indicate that small numbers of ON-REP cells are sufficient for recovery from anemia because EPO-production levels in each ON-REP cell are very high.

Whereas the origins of myofibroblasts coming into existence in the fibrotic kidneys of CKD patients are controversial and considered various (LeBleu et al., 2013), we and others have demonstrated that resident interstitial fibroblasts, including REP cells in healthy kidneys, are transformed into myofibroblasts under pathological conditions (**Figures 1C, D**; Humphreys et al., 2010; Asada et al., 2011; Souma et al., 2013). Importantly, REP cells gain proliferative activity and lose EPO-production ability after transformation (Souma et al., 2013). Thus, REP cells are closely related to the two major pathologies of CKD: renal anemia and fibrosis. Therefore, investigations of REP cells and myofibroblast-transformed REP (MF-REP) cells hold the key to elucidating the molecular pathology of CKD.

Various studies have proposed that the transformation of REP cells into MF-REP cells is promoted by the SMAD and NFκB transcription factors, which are activated by transforming growth factor beta (TGFβ) and tumor necrosis factor alpha, respectively (Wynn and Ramalingam, 2012; Souma et al., 2015). Additionally, DNA methylation in the *EPO*-gene promoter is thought to be involved in the loss of EPO-production ability in MF-REP cells (Chang et al., 2016). To further elucidate the molecular pathology of CKD by characterizing MF-REP cells, we recently established a myofibroblast cell line derived from mouse REP cells, and the cell line was referred to as Replic (REP cell-lineage immortalized and cultivable) cells (Sato et al., 2019). The genomic region of the *EPO*-gene promoter is highly methylated in Replic cells, and cell-autonomous TGFβ signaling supports their myofibroblast properties, which include the expression of genes for α smooth muscle actin, fibronectin, and collagens, among others.

## *EPO*-Gene Regulation in REP Cells

EPO production in REP cells is strictly regulated at the gene transcription level, and transcription is likely regulated by an ON/OFF mechanism in each cell (Obara et al., 2008). Gene expression data of separately isolated ON- and OFF-REP cells indicated that hypoxia-inducible genes are highly expressed in ON-REP cells compared to OFF-REP cells, suggesting that there is a hypoxic threshold to activate *EPO*-gene expression in REP cells and that the oxygen levels of the microenvironments around ON-REP cells are below the threshold (Yamazaki et al., 2013). The expression levels of almost all hypoxia-inducible genes, including genes related to angiogenesis, glycolysis, and cell survival, are commonly regulated by hypoxia-inducible transcription factors (HIFs) (**Figure 1E**; Wang and Semenza, 1993; Lendahl et al., 2009; Suzuki et al., 2017).

HIFs consist of two subunits, namely, HIFα and HIFβ (also known as ARNT), and they bind to specific DNA sequences (^A^/_G_CGTG) in the regulatory regions of their target genes (Semenza et al., 1991; Lendahl et al., 2009; Haase, 2013). Under normal air conditions (normoxia), specific prolyl residues of HIFα are hydroxylated with HIF-specific prolyl hydroxylase domain proteins (PHDs) by means of intracellular oxygen, and hydroxylated HIFα proteins are degraded by the ubiquitin-proteasome system (**Figure 1E**; Lendahl et al., 2009). In cells with insufficient oxygen for PHD-mediated HIFα hydroxylation, HIFα proteins avoid degradation and activate transcription of their target genes. There are three isoforms encoded by the different genes for the PHD and HIFα proteins, respectively. Among the isoforms, PHD2 and HIF2α primarily control *EPO*-gene expression in a hypoxia-inducible manner in REP cells (**Figure 1D**; Castrop and Kurtz, 2010; Souma et al., 2016). Therefore, dysfunction of the PHD2-HIF2α-*EPO* axis in REP cells is considered the molecular cause of renal anemia. Notably, polycythaemia-related polymorphisms are found in the genes for PHD2 and HIF2α but not in those for the other isoforms, and these polymorphisms are predicted to lead to HIF2α stabilization followed by *EPO*-gene induction without hypoxic stimuli (Bento et al., 2014).

Due to the difficulty of isolating sufficient levels of REP cells for molecular biology analyses, hepatocytes and genetically modified mice have been used for studies on *EPO*-gene regulation. With transgenic mouse strategies, the murine *Epo*-gene regulatory region for REP-cell-specific and hypoxia-inducible expression was determined to be approximately 10 kb upstream from the transcription start site of the *Epo* gene (Hirano et al., 2017). We also discovered that histones located in the *EPO*-gene promoter are always acetylated regardless of hypoxic EPO induction and that histones are dissociated from the nucleosome structure in the *EPO*-gene promoter of hepatocytes under hypoxic conditions through HIF2α activation (Suzuki et al., 2011; Tojo et al., 2015). Nucleosome disassembly results in the formation of a nucleosome-free region (NFR) that has an open chromatin structure for the direct association between transcription factors and promoters and allows the induction of *EPO*-gene transcription (**Figure 1E**; Suzuki et al., 2017; Suzuki et al., 2018a).

## Stepwise Mechanisms of *EPO*-Gene Silencing in MF-REP Cells

Since mice lacking PHD2 expression in REP cells are resistant to renal EPO deficiency caused by kidney injury, inappropriate over-activation of PHD2 is considered responsible for *EPO*-gene inactivation in MF-REP cells (**Figure 1D**; Souma et al., 2016). Although the oxygen affinities of PHDs are ordinarily very low compared to those of other oxygen-dependent enzymes, including collagen hydroxylases and epigenetic regulators (see below; Hancock et al., 2017; Chakraborty et al., 2019), unknown mechanisms are speculated to allow PHDs to use oxygen in MF-REP cells even under pathological hypoxic conditions. Indeed, PHD inhibitory compounds are being developed as medicines for renal anemia treatment, and clinical trials of these compounds are showing anticipated effects (**Figure 1D**; Akizawa et al., 2019).

In addition to PHD over-activation, DNA methylation in the *EPO* promoter is involved in *EPO*-gene silencing in MF-REP cells (Chang et al., 2016; Sato et al., 2019). Because hyper-methylation of gene promoter regions blunts gene transcription by tightly compacting the chromatin structure and blocking associations with transcription factor complexes (Jones, 2012; Schübeler, 2015), PHD inhibitors are predicted to be ineffective in cells in which the *EPO* promoter is highly methylated (**Figure 1D**). Consistent with this hypothesis, in Replic cells, neither PHD inhibitors nor HIF2α overexpression activated the *Epo* gene, which is highly methylated (Sato et al., 2019). Thus, the transformation of REP cells into myofibroblasts is divided into at least two consecutive stages: the early MF-REP (eMF-REP) cell stage with over-activation of PHD and the progressive MF-REP (pMF-REP) cell stage with hyper-methylation of the *EPO* promoter. PHD inhibitors are theoretically effective at inducing EPO production in the former cell type but ineffective in the latter cell type, which likely corresponds to Replic cells. Intriguingly, transformation of REP cells is reversible in the early stages of kidney injury (**Figure 1D**; Souma et al., 2013).

We recently discovered that the gene promoter for HIF2α is also highly methylated and that both the mRNA and protein of HIF2α are undetectable in pMF-REP cells, even under hypoxic conditions (Sato et al., 2019). This finding indicates that DNA methylation in specific gene promoters is one of the causes of EPO deficiency in CKD. DNA methylation is mediated by 3 DNA methyltransferases (DNMTs): DNMT1, DNMT3A, and DNMT3B. DNMT1 is essential for the maintenance of DNA methylation patterns beyond mitosis to inherit epigenetic memory (Jeltsch, 2006), while *de novo* DNA methylation is mediated by DNMT3A and DNMT3B (Hsieh, 1999). This transformation enhances the expression of mRNAs for DNMT1 and DNMT3B by TGFβ signaling (Souma et al., 2013), suggesting that these DNMTs are involved in the loss of EPO-production ability in MF-REP cells. In fact, 5-aza-2’-deoxycytidine (5-aza), an inhibitor of DNMT1, restores EPO production in primary-cultured mouse MF-REP cells by reducing DNA methylation in the *Epo*-gene promoter (Chang et al., 2016). In contrast, DNA methylation in the gene promoters for EPO and HIF2α in Replic cells was resistant to 5-aza treatment, whereas the other genomic regions tested were sensitive (Sato et al., 2019). This discrepancy in 5-aza efficacy between the primary-cultured MF-REP cells and Replic cells, which may represent eMF-REP and pMF-REP cells, respectively, is explained by differences in the activity of *de novo* DNA methylation because expression of *de novo* DNMTs (DNMT3A and DNMT3B) is induced by TGFβ signaling (Cardenas et al., 2014), which is autonomously promoted in Replic cells.

## Efficacy of PHD Inhibitors in EPO-Induction Is Related to the Transformation Stage of Myofibroblast-Transformed REP Cells

As an alternative to ESAs, PHD inhibitors are a promising group of next-generation medicines for renal anemia treatment because they are orally administrable small compounds (Martin et al., 2017). The first PHD inhibitor, roxadustat, was launched in 2018 in China, where there are more than 100 million CKD patients (Zhang et al., 2012). However, it is concerned that PHD inhibitors cause unexpected side effects through their widespread activation of HIF-target genes in addition to *EPO*, and these genes include genes that are related to energy metabolism, angiogenesis, and cell survival (Wang and Semenza, 1993). Although obvious adverse events, such as tumor malignancy, have not been observed in clinical trials thus far (Akizawa et al., 2019), further long-term observation is necessary to confirm both the beneficial and unfavorable side effects of PHD inhibitors.

PHDs catalyze oxygenation reactions of the specific prolyl residues of HIFαs to produce hydroxylated HIFαs using oxygen, iron, ascorbate, and α-ketoglutarate. These substrates are also used by a variety of α-ketoglutarate-dependent dioxygenases, including important epigenetic regulators, TET (ten-eleven translocation) family DNA demethylases and KDM (histone lysine demethylase) family histone demethylases (Itoh et al., 2013; Kohli and Zhang, 2013). Since PHDs show the lowest affinity for oxygen among these dioxygenases, PHDs are the first dioxygenases inactivated by hypoxia and can thus sensitively detect hypoxia in cells. On the other hand, the other dioxygenases are less susceptible to hypoxia than PHDs. Notably, very recent studies have shown that some KDMs are as sensitive to hypoxia as PHDs, and further studies are expected to unveil mechanisms involving direct sensing of hypoxia by epigenetic regulators in addition to PHDs (Batie et al., 2019; Chakraborty et al., 2019). Since the current PHD inhibitors commonly block the specific association of α-ketoglutarate with PHDs, other α-ketoglutarate-dependent dioxygenases are unresponsive to these compounds.

In summary, PHD inhibitors are considered to be effective in eMF-REP cells but not in pMF-REP cells with methylation-based silencing of the genes for HIF2α and EPO (**Figure 1D**). Our preliminary experiments using mouse models have shown that EPO production is induced by PHD inhibitors in undamaged or slightly damaged REP Cells of fibrotic kidneys through HIF2α accumulation but not in severely damaged areas. In contrast, PHD inhibitors activate EPO production in almost all the REP cells of healthy kidneys within 6 H after peritoneal injection of the drug (Suzuki et al., 2018b). Clinical trials have demonstrated that PHD inhibitors induce erythropoiesis in nephric patients suffering from any CKD stage and end-stage renal disease, but anephric patients barely respond to PHD inhibitors with regard to EPO induction (Bernhardt et al., 2010). Taken together, these results suggest that EPO produced by a small number of REP cells in the kidney is sufficient to induce erythropoiesis in renal anemia patients. Indeed, as mentioned above, ON-REP cells constitute less than 10% of the REP cells in mouse models of severe chronic anemia (**Figure 1C**; Yamazaki et al., 2013). These observations also suggest that the efficacy of PHD inhibitors differs among renal anemia patients and that the population of eMF-REP and healthy REP Cells in each patient defines their responsiveness to PHD inhibitors.

## Perspectives: Non-Invasive Strategies for Personalized Precision Medicine for Chronic Kidney Disease

Here, we summarize the epigenetic and molecular mechanisms of *EPO*-gene silencing in CKD patients and propose the stepwise transformation of REP cells into eMF-REP and pMF-REP cells in injured kidneys (**Figures 1C, D**). We also suggest that PHD-inhibitor responsiveness varies in patients and is dependent on the degree of REP cell transformation, which fundamentally correlates with the degree of kidney fibrosis in CKD. Thus, diagnosing the degree of kidney fibrosis is expected to inform us not only about CKD conditions/prognoses but also about PHD-inhibitor responsiveness of CKD patients. Currently, an invasive biopsy is widely adopted for the diagnosis of the complicated pathology of CKD (Mise et al., 2014). However, non-invasive biomarkers for the progression of CKD are being explored. For example, urine concentrations of N-acetyl-β-D-glucosaminidase (Bazzi et al., 2002) can be used. However, the quantitative relationship of the biomarkers to the degree of kidney fibrosis should be investigated in detail.

We propose that urine exfoliated cells can be used for the diagnosis and prediction of CKD. Urine contains several types of kidney cells, including tubular epithelial cells and podocytes, which are living and proliferative in *ex vivo* culture (Dörrenhaus et al., 2000; Kumagai et al., 2000; Vogelmann et al., 2003; Oliveira Arcolino et al., 2015). Therefore, these cells have been utilized as the experimental source of human renal epithelial cells and investigated as biomarkers for the early detection of bladder cancer (Rahmoune et al., 2005; Shimizu et al., 2013). Additionally, urine from CKD patients contains more cultivable exfoliated cells than urine from healthy individuals, which is advantageous for diagnosis (Detrisac et al., 1983). Importantly, our preliminary RT-PCR experiments detected the expression of mRNAs for EPO, HIF2α, and CD73 in cultured cells from the urine of patients with kidney disease, indicating that the exfoliated cell cultures contain REP cells and/or MF-REP cells. In addition, REP cells and MF-REP cells can be purified from the mixtures of exfoliated cells with cell surface expression of CD73 or PDGFRβ using cell sorters (Armulik et al., 2011; Pan et al., 2011).

With small numbers of urine exfoliated cells, high-sensitivity PCR-based techniques are expected to detect HIF2α mRNA expression and *EPO*-gene methylation. NFRs are also detectable with PCR, as we have identified NFRs in hypoxia-inducible gene promoters (Tojo et al., 2015; Suzuki et al., 2018a). Taking advantage of living cells, drug sensitivity may be directly investigated in urine exfoliated cells. Although further studies are needed, exfoliated cells in urine would provide novel diagnostic strategies to distinguish pMF-REP and eMF-REP for the prediction of PHD-inhibitor responsiveness, as well as plausible biomarkers for kidney fibrosis and CKD prognosis.

## Author Contributions

NS conceived the idea. KS, NK, and NS wrote the manuscript and created the figures.

## Funding

This work was supported in part by Takeda Life Science Foundation and SENSHIN Medical Research Foundation (for NS). The funders have no role in this study.

## Conflict of Interest

The authors declare that the research was conducted in the absence of any commercial or financial relationships that could be construed as a potential conflict of interest.
